# Lower anxiety level to perform movements after revision anterior cruciate ligament reconstruction with lateral extra-articular tenodesis compared to without lateral extra-articular tenodesis

**DOI:** 10.1007/s12306-024-00818-0

**Published:** 2024-05-01

**Authors:** T. Vendrig, M. N. J. Keizer, R. W. Brouwer, H. Houdijk, R. A. G. Hoogeslag

**Affiliations:** 1 University Medical Center Groningen, Center for Human Movement Sciences, University of Groningen, UMCG Sector F, FA 23, PO Box 196, Groningen, 9713 AV The Netherlands; 2 Department of Orthopedic Surgery, Martini Hospital, Groningen, Groningen, The Netherlands; 3 Centre for Orthopaedic Surgery and Sports Medicine OCON, Hengelo, The Netherlands

**Keywords:** Revision ACL reconstruction, Lateral extra-articular tenodesis, Iliotibial band, Anxiety, Fear of reinjury, Kinesiophobia

## Abstract

**Purpose:**

To evaluate the anxiety level to perform movements in patients after revision anterior cruciate ligament reconstruction (ACLR) combined with lateral extra-articular tenodesis (LET) compared to patients after revision ACLR without LET.

**Methods:**

Ninety patients who underwent revision ACLR with ipsilateral bone-patellar tendon-bone autograft and with a minimum of 12 months follow-up were included in this study. Patients were divided into two groups: patients who received revision ACLR in combination with LET (revision ACLR_LET group; mean follow-up: 29.4 months, range: 12–80 months), and patients who received revision ACLR without LET (revision ACLR group; mean follow-up: 61.1 months, range: 22–192 months). All patients filled in a questionnaire about anxiety level related to physical activity and sports, the Knee injury and Osteoarthritis Outcome Score (KOOS), the International Knee Documentation Committee subjective form (IKDC_subjective_), and the Tegner Activity Score.

**Results:**

Patients in the revision ACLR_LET group had a significantly lower anxiety level to perform movements than patients in the revision ACLR group (*p* < 0.05). No significant differences were found in KOOS, IKDC_subjective_, and Tegner Activity Scores.

**Conclusions:**

Patients who received LET in addition to revision ACLR have a lower anxiety level to perform movements than patients with revision ACLR alone, despite non-different subjective functional outcomes.

**Study design:**

Retrospective cohort study, Level of evidence: III.

## Introduction

Anterior cruciate ligament (ACL) injuries are commonly treated with ACL reconstruction (ACLR) [22]. However, 7% to 18% of patients who undergo an ACLR sustain graft failure [27, 28]. A secondary ACL injury is usually treated with revision ACLR, but this leads to inferior functional outcomes compared to primary ACLR [8]. Seventy-nine percent of patients report to have anxiety concerning reinjuring their knee after revision ACLR, which could be a factor leading to inferior outcomes [16]. On the other hand, a factor that could increase anxiety is the presence of residual rotational instability after ACLR [25].

Structures in the anterolateral corner of the knee contribute positively to rotational stability [1, 2]. Anterolateral corner injury is a common secondary injury in ACLR patients [5]. Several surgical procedures to stabilize the anterolateral corner, such as a lateral extra-articular tenodesis with the iliotibial band (LET) are reported to be beneficial for rotational stability in ACLR patients [13]. In fact, a decrease in rotational instability together with an increase in subjective outcomes after primary ACLR combined with anterolateral corner reconstruction has been reported [7, 24]. In patients with revision ACLR an additional LET can also be beneficial, as the return to sports rate is higher in patients who received revision ACLR combined with LET compared to patients who only received revision ACLR [14]. Fear of reinjury is the most common reason for not returning to sports [19], so a possible reason for the higher return to sports rate is that patients with an ACLR and additional LET have a lower anxiety level. However, the actual influence on the anxiety level in performing movements after revision ACLR with LET compared to revision ACLR without LET is unknown.

Therefore, the aim of the current study was to evaluate the anxiety level to perform movements in patients after revision ACLR combined with LET compared to patients after revision ACLR without LET. Additionally, functional outcome scores were evaluated among the two groups. It is hypothesized that patients who received a revision ACLR combined with LET show lower anxiety levels to perform movements and better functional outcome scores than patients who received revision ACLR without LET.

## Methods

**Fig. 1:**
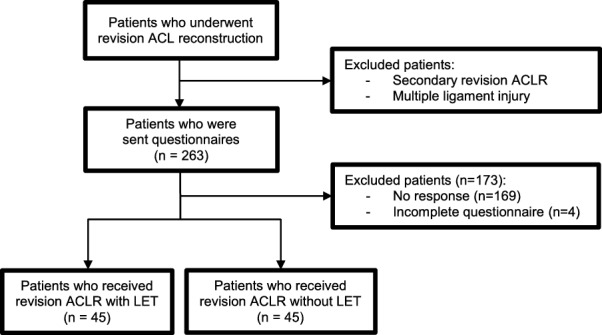
Flowchart of included patients

A retrospective cohort study was conducted at the Centre for Orthopaedic Surgery and Sports Medicine OCON in Hengelo, The Netherlands and at the Martini Hospital in Groningen, The Netherlands. The institutional review board of Centre for Orthopaedic Surgery and Sports Medicine OCON approved this study (IRB nr: 2,020,101). Patients who received revision ACLR with an ipsilateral bone-patellar tendon-bone (BPTB) autograft, with a minimum follow-up of twelve months, and played a pivoting sport before injury were eligible for this study (Fig. 1). Exclusion criteria were additional knee surgery after revision ACLR and a history of contralateral ACL injury. After inclusion, patients were divided into two groups: 1) patients who received revision ACLR in combination with LET (revision ACLR_LET group), and 2) patients who received revision ACLR without LET (revision ACLR group). The difference in surgical procedure between groups was the result of a change in protocol over the years, when the involved surgeons switched to additional LET based on emerging evidence. High knee instability or other patient related factors such as gender were not a factor for receiving additional LET or not.

### Outcome measures

The outcome measures were:

- The anxiety level of patients to perform certain movements, on a scale of 1 to 10 (10 being the highest anxiety level).
- The Dutch version of the Knee injury and Osteoarthritis Outcome Score (KOOS) [4].
- The International Knee Documentation Committee subjective form (IKDC_subjective_) [10].
- The Tegner Activity Score [26].

### Data collection

All included patients were asked to fill in a self-designed questionnaire, which contained questions about anxiety level to perform movements [15], the KOOS subscales, IKDC_subjective_, and Tegner Activity Score. The specific question to examine the anxiety level to perform movements was: “*Does your knee injury affect you in such a way that you are anxious to perform certain actions and if so how anxious are you (1–10)?*” [15]. This questionnaire was sent to the patients by e-mail, together with an explanation of the study. The patients gave their informed consent by submitting the questionnaire. After receiving the filled in questionnaire, the researcher obtained baseline characteristics from the patients’ file, the graft type used for primary ACLR and information about accompanying meniscal or cartilaginous injuries from the patients’ operative form.

### Surgical technique

All surgeries were performed by two experienced orthopaedic surgeons (RAGH and RWB). The surgeons performed one-stage (n = 80) or two-stage (n = 10) ACLR revision surgery, depending on tunnel positioning and/or widening, for which bone-grafting was necessary. ACLR revision was performed using a BPTB autograft harvested from the ipsilateral leg. For drilling the femoral socket the anteromedial portal technique was used. The ACL graft was fixed with interference screws. The modified deep Lemaire technique was used to perform LET [13, 18], using an interference screw to fix the reconstruction [12].

### Statistical analysis

The data were processed and statistical testing was performed using SPSS version 26 (IBM SPSS Statistics for Windows, Version 27.0. Armonk, NY: IBM Corp). Independent sample t-test was used to compare the distribution of anxiety level (1–10) between groups (data was continuous and was normally distributed). Fischer’s exact test was used to compare the movements during which anxiety was reported between groups. Independent sample t-tests were used to compare the scores on the KOOS subscales and Tegner Activity Score between groups. To compare the KOOS_ADL_ subscale and IKDC_subjective_ between groups the Mann–Whitney U tests was used because of the highly skewed data (skewness < -2). An alpha level of p ≤ 0.05 was considered statistically significant.

A post-hoc power calculation using means of 4.3 (3.1) and 2.8 (2.6), an alfa of 0.05, and group sizes of 45, revealed a power of 70% for analysis regarding the anxiety level calculated using G*Power 3.1.

## Results

### Baseline characteristics

Ninety patients were included for analysis, with a mean follow-up of 45.3 months (range follow-up: 12–192 months; Table 1). The difference in follow-up time between groups was the result of a change in protocol over the years, when the involved surgeons switched to additional LET based on emerging evidence.

**Table 1:** Baseline characteristics of the analyzed patients

	Revision ACLR group^a^	Revision ACLR_LET group^b^	*P* value
Number of patients	45	45	
Male/female	31/14	35/10	n.s
Age^c^ [mean (SD)]	29.6 (7.7)	26.4 (6.78)	n.s
Left/right knee	21/24	20/25	n.s
Follow-up period (months) [mean (range)]	61.1 (22–192)	29.4 (12–80)	< .001*
One-/two stage revision	43/2	37/8	n.s
Primary graft used *Hamstring autograft*	45	45	n.s
Cartilage injury			n.s
*Medial*	21	12	
*Lateral*	12	10	
*Patellar*	6	7	
Medial meniscal injury			n.s
*No*	18	27	
*Yes, no treatment*	3	2	
*Yes, meniscectomy*	19	13	
*Yes, meniscus repair*	5	3	
Lateral meniscal injury			n.s
*No*	31	26	
*Yes, not treatment*	2	6	
*Yes, meniscectomy*	12	13	

^*^Significant

^a^Patients who received revision anterior cruciate ligament reconstruction without a lateral extra-articular tenodesis

^b^Patients who received revision anterior cruciate ligament reconstruction with a lateral extra-articular tenodesis

^c^At follow-up

### Anxiety level and functional outcomes

Patients in the revision ACLR group had a significantly higher anxiety level to perform movements than patients in the revision ACLR_LET group (t(88) = 2.44; p < 0.05; Table 2). Movements during which anxiety was reported are presented in Table 3, demonstrating no statistically significant difference in anxiety in performing specific movements between groups (p > 0.05). No statistically significant differences were found between the revision ACLR group and revision ACLR_LET group in KOOS subscales, IKDC_subjective_, and Tegner Activity Score (Table 2).

**Table 2:** Anxiety level, KOOS, Tegner activity score and IKDC_subjective_ with a minimum follow-up of one year after surgery

	Revision ACLR^a^	Revision ACLR_LET^b^	*P* value (2-tailed)
Anxiety level [mean (SD)]	4.3 (3.1)	2.8 (2.6)	.02*
KOOS_pain_ [mean (SD)]	89.07 (12.8)	90.2 (10.3)	n.s
KOOS_symptoms_ [mean (SD)]	62.1 (12.9)	60.2 (11.0)	n.s
KOOS_sport_ [mean (SD)]	68.9 (22.9)	72.0 (18.1)	n.s
KOOS_quality of life_ [mean (SD)]	54.5 (15.8)	53.9 (13.2)	n.s
KOOS_activities of daily living_ (median)	98.5	99.0	n.s
Tegner Activity Score [mean (SD)]	5.62 (2.5)	4.9 (2.6)	n.s
IKDC_subjective_ (median)	82.9	82.9	n.s

^*^Significant

^a^Patients who received revision anterior cruciate ligament reconstruction without a lateral extra-articular tenodesis

^b^Patients who received revision anterior cruciate ligament reconstruction with a lateral extra-articular tenodesis

IKDC = International Knee Documentation Committee, KOOS = Knee injury and Osteoarthritis Outcome Score

**Table 3:** Self-reported reasons for anxiety

Movements during which anxiety is reported	Revision ACLR group^a^ (n)	Revision ACLR_LET group^b^ (n)
Sport-related activities	17	23
Jumping and landing	12	9
Tossing and turning	6	10
Sudden movements	2	1
Running	2	0
Carrying heavy loads	1	0
Kneeling	1	0
Squatting	0	1
Unstable movements	0	2

Fisher’s exact test reported no significant difference between groups (*p* > .05)

^a^Patients who received revision anterior cruciate ligament reconstruction without a lateral extra-articular tenodesis

^b^Patients who received revision anterior cruciate ligament reconstruction with a lateral extra-articular tenodesis

## Discussion

The most important finding of this study is that patients after revision ACLR with additional LET experienced a lower anxiety level to perform movements than patients after revision ACLR without LET. Knee instability is reported to negatively affect the physical functioning of the knee [6]. Poorer physical functioning of the knee is related to higher anxiety [23]. Additionally, the perception of moderate to severe knee instability is associated with greater knee pain [3], which is related to the amount of pain-related fear a patient experiences [11]. Moreover, increased knee instability may lower confidence in a patient's knee, which is associated with higher fear of reinjury [9]. Therefore, a possible explanation for the lower anxiety level to perform movements in patients with an additional LET might be a reduction in rotational instability of the knee [7]. Another possible explanation could be that patients who received additional LET received positive psychological reinforcement due to the fact that an extra procedure was performed in comparison to the primary ACLR. This could have given the patients with additional LET more confidence in their knee, which is associated with lower fear of reinjury [9].

Lowering the anxiety level to perform movements in patients after revision ACLR is relevant for multiple reasons. Patients with greater fear are more likely to report lower levels of activity [20]. Secondly, a review of Nwachukwu et al. reported that the most common reason for not returning to sports was fear of reinjury [19]. Additionally, greater fear of movement and reinjury are correlated with lower health-related quality of life [21]. Orthopaedic surgeons should take these factors in consideration for the treatment of patients with a failure of a primary ACLR.

This current study found no difference in the functional outcome of the knee on the basis of the KOOS, IKDC_subjective_, and Tegner Activity Score between patients with a revision ACLR and LET compared to patients with only a revision ACLR. This is in line with the study of Keizer et al. [14]. Getgood et al. also found no differences in all KOOS domains and IKDC_subjective_ score after 12 and 24 months [7]. However, the study of Getgood et al. was done with patients after primary ACLR instead of revision ACLR [7]. The study of Sonnery-Cottet et al. also found no difference in Tegner Activity Score between patients with primary ACLR combined with anterolateral reconstruction and patients with only primary ACLR [7]. In contrast to this current study, the results of Sonnery-Cottet et al. showed a better KOOS and IKDC_subjective_ score in patients with primary ACLR and additional anterolateral reconstruction compared to patients without additional anterolateral reconstruction [24]. However, the study of Sonnery-Cottet et al. used patients with primary ACLR instead of revision ACLR, and used a hamstring tendon autograft for anterolateral reconstruction instead of LET [24].

Besides the limitations that are inherent to all retrospective studies, some other limitations have to be discussed. Due to a philosophy change, all revision ACLR patients received additional LET at a certain point. Although this resulted in two similar patient groups, it also resulted in a significantly shorter follow-up of patients with additional LET. The physical function of the knee increases when time goes by after surgery [17]. So, with a shorter follow-up period you would expect worse functional outcome scores, but the results of this study show similar physical function. If the follow-up duration of the ACLR_LET group would have been longer, the functional outcome scores could have been significantly higher in the ACLR with LET group, but more research is necessary to conclude this. Additionally, it is possible that the difference in anxiety level would have been larger with an equal follow-up period. Another limitation is that no objective measurement of knee instability was done. This could have provided additional data to support the hypothesis that the lower anxiety level of patients with additional LET is possibly the result of reduced rotational knee instability. Furthermore, it should be noted that the post-hoc power analysis showed a relatively low power of 70%. Lastly, no proven reliable anxiety questionnaire was used. However, this study does provide insight into the feeling of anxiety patients experience to perform movements.

## Conclusions

Our study provides evidence that the anxiety level to perform movements is lower in patients who received revision ACLR combined with LET compared to patients who only received revision ACLR in our cohort, despite non-different subjective functional outcomes. This might be a reason for orthopaedic surgeons to consider additional LET with revision ACLR for patients with failure of a primary ACLR. A randomized controlled trial should confirm these findings.

## Data Availability

The dataset of the current study is not publicly available due to privacy reasons but are available from the corresponding author on reasonable request.

## Abbreviations

ACL: Anterior cruciate ligament
ACLR: Anterior cruciate ligament reconstruction
BPTB: Bone-patellar tendon-bone
IKDC: International knee documentation committee
KOOS: Knee injury and osteoarthritis outcome score
LET: Lateral extra-articular tenodesis with the iliotibial band
revision ACLR_LET group: Patients who received revision ACLR combined with LET
revision ACLR group: Patients who only received revision ACLR
